# Deviations in palatal region between indirect and direct digital models: an in vivo study

**DOI:** 10.1186/s12903-019-0751-3

**Published:** 2019-04-27

**Authors:** Yang Zhongpeng, Xu Tianmin, Jiang Ruoping

**Affiliations:** 1 National Engineering Laboratory for Digital and Material Technology of Stomatology, Peking University School and Hospital of Stomatology, No.22 Zhongguancun South Avenue, Haidian District, Beijing, 100081 China; 2 Department of Orthodontics, Peking University School and Hospital of Stomatology, No.22 Zhongguancun South Avenue, Haidian District, Beijing, 100081 China

**Keywords:** Intraoral scan, Digital model, Palatal soft tissue, Model superimposition, Trueness

## Abstract

**Background:**

Studies focusing on accuracy of intraoral digital models in the palatal region are scarce. The present study aimed to investigate the influence of different scanning sequences on palatal trueness and to assess deviation and distribution character of trueness in palate.

**Methods:**

Overall, 35 participants accepted three types of procedures to acquire upper digital models. Indirect models digitalised from plaster models were considered as the reference. Two direct digital models were acquired using TRIOS 3 POD intraoral scanners, namely Groups Tr1 and Tr2, wherein intraoral scanning differed in terms of palatal scanning sequences. Based on a modified dental-level superimposition method, 3D measurements of trueness in palate and palatal vault region (PVR) for palatal stable regional superimposition in Groups Tr1 and Tr2, respectively, were performed. Absolute deviations were measured for trueness, while signed deviations were analysed for shape distortion. Colour-coded maps were used for quantitative analysis of deviation distribution pattern. Paired t test was used to analyse differences in palatal trueness between different scanning sequences. One-way repeated-measures analysis of variance and Bonferroni test were used to compare trueness measurements among different superimposition methods. Intraclass correlation coefficient (ICC) was used to verify reproducibility of the proposed method.

**Results:**

Palatal trueness in Group Tr1 (118.59 ± 37.67 μm) was slightly less accurate than that (108.25 ± 33.83 μm) in Group Tr2 (*p* = 0.012 < 0.05). Trueness of PVR in Groups Tr1 (127.35 ± 54.11 μm) and Tr2 (118.17 ± 49.52 μm) did not differ significantly (*p* = 0.149). Moreover, no significant difference was noted in distortion of the palatal region and PVR in Groups Tr1 and Tr2 (*p* = 0.582 and 0.615, respectively). A similar pattern of palatal trueness was noted in a majority of participants (22/35). For 3D palatal trueness measurement, there were different applications for different superimposition methods. ICC for the proposed method was > 0.90.

**Conclusions:**

Scanning sequences can affect palatal trueness. Palatal scanning should be initiated at the palatal side of the posterior teeth where the initial scan begins. For 3D PVR superimposition, distal boundary of the selected region should be adjusted mesially whilst referring to intraoral digital models.

**Trial registration:**

The trial has been registered (registration No: R000039467, Trial ID: UMIN000034617, date of registration: 2018/10/24‘retrospectively registered’).

## Background

Dental treatment of any type usually necessitates evaluation of the intraoral situation. With introduction of intraoral scanners in dentistry, direct acquisition of digital impressions is gaining popularity [1] as it provides advantages of reducing the number of procedures required as well as storage space. Several studies have investigated the accuracy of intraoral scans, which encompasses two parameters, namely ‘trueness’ and ‘precision’ [2–5]. Numerous factors can affect accuracy of intraoral scans; typically, these can be categorised into three types—scanning principles [6], scanning environment [7, 8] and process of data synthesis [6, 7, 9–14]. When performing a direct scan of the oral environment, trueness can be > 200 μm [7], while precision can be > 1000 μm [8]. Moreover, attention must be paid to scanning strategies used since these strategies have a marked influence on accuracy and distribution of deviations in full-arch intraoral scans [11–13]. Larger deviations are observed in regions wherein continuous full-arch scanning is terminated [12]. Since it is not possible to change scanning principles or avoid oral environment factors, application of an appropriate scanning strategy may help improve the accuracy of intraoral scanning.

Most studies on accuracy have focused on dental hard tissues, ranging from single tooth crown to full arches [2–12, 15]. Irrespective of statistical differences between intraoral digital and conventional impressions, direct digital dental model is clinically acceptable [1–4]. However, much less attention is paid to soft tissue accuracy of intraoral digital models. Numerous appliances used in prosthodontics and orthodontics need to cover certain parts of the palatal region, such as removable partial dentures, obturators, retainers and Hyrax appliances [16–19]. In addition, characteristics of the palatal region can be utilised to study human identification, articulometry and the impact of oral habits [20–22]. Furthermore, because of multiple features of palatal rugae and the sufficient area of the entire palatal region, dental changes of orthodontic treatment are usually evaluated using 3D superimposition in a specific palatal region [23–25]. By using stationary mini-screws as a reference, Chen et al. (2011) [25] found a reliable palatal region with 500-μm deviations for assessing dental changes in adults. This method is called ‘3D palatal vault regional (PVR) superimposition’, which is now widely used in clinical practice. Therefore, owing to non-substitutability of the palatal region in various clinical fields, it is essential to identify deviation and its distribution of accuracy in intraoral palatal scanning.

Nevertheless, the soft tissue accuracy of intraoral scanning has not been validated yet owing to scarcity of relevant studies. According to an in vivo study by Gan et al. (2016) [10], trueness of digital impression acquired by TRIOS POD scanners in the palatal region (130.54 ± 33.95 μm) was less accurate than that in the dentition (80.01 ± 17.78 μm). A similar trueness of intraoral palatal scans was identified by Deferm et al. (2018) [26]. Moreover, to our knowledge, there is no research focusing on the stable palatal region for superimposition of intraoral scanning.

To our knowledge, to date, none of studies on scanning strategies have focused on palatal soft tissues. Even the User Guide (3 Shape, Denmark) does not provide any specific description of palatal scanning strategy. Therefore, the effect of scanning sequences on the palatal region should be clarified so that it would be clear to estimate the region displaying a higher deviation in terms of scanning sequences.

To date, the two most relevant studies on 3D measurement of accuracy of intraoral scanning in the palatal region have used surface-based registration between intraoral digital models and digitalised plaster models by using a best-fit algorithm [10, 26]. Deferm et al. (2018) [26] measured palatal accuracy by using model superimposition at a full-arch level. While the global superimposition method was used in the other study [10], the details about the selected area for superimposition were unclear. Since the accuracy of intraoral digital impression on dentition has been verified as substantially high [1–4], dentition can be used for surface-based registration to measure accuracy in the palatal region. However, intraoral scans appear to have greater divergences in certain specific dental regions, particularly in the distal end of the arch, the anterior region and interproximal surfaces [2–4, 8, 12, 14]. Moreover, gypsum casts from polyvinyl siloxane (PVS) impressions showed increasing deviations of both trueness and precision at the distal end of the arch [3]. Therefore, in order to avoid relatively less accurate dental regions and to involve more surface characters, a modified dental-level superimposition method was proposed in this study.

Aims of this in vivo study were (i) to compare accuracy of 3D measurement of intraoral palatal scans among different superimposition methods; (ii) to investigate the influence on trueness of intraoral palatal scans by using different scanning sequences; and (iii) to assess deviation and its distribution character of trueness in the palatal and PVR region.

## Methods

### Participants

Thirty-seven students from the School of Stomatology were recruited in this study. Eventually, 35 volunteers successfully finished entire data acquisition. Informed consent was obtained from all participants.

Inclusion criteria were as follows:

1. Age between 18 and 30 years;
2. Permanent dentition, including premolars symmetrically extracted for past orthodontic treatment with retention stage ended;
3. Good oral hygiene; and
4. Good cooperation to finish all data acquisition.

Exclusion criteria were as follows:

1. Undergoing orthodontic treatment or finished orthodontic treatment for no more than 2 years;
2. With more than one-third of tooth defect on a single tooth;
3. With large amounts of metal restorations;
4. With severe crowding or obvious spaces;
5. With palatal defect or lesion;
6. With an abnormally large arch width between bilateral upper first molars; and
7. Moderate to severe periodontitis or obvious gingivitis.

### Digital model acquisition

**Fig. 1:**
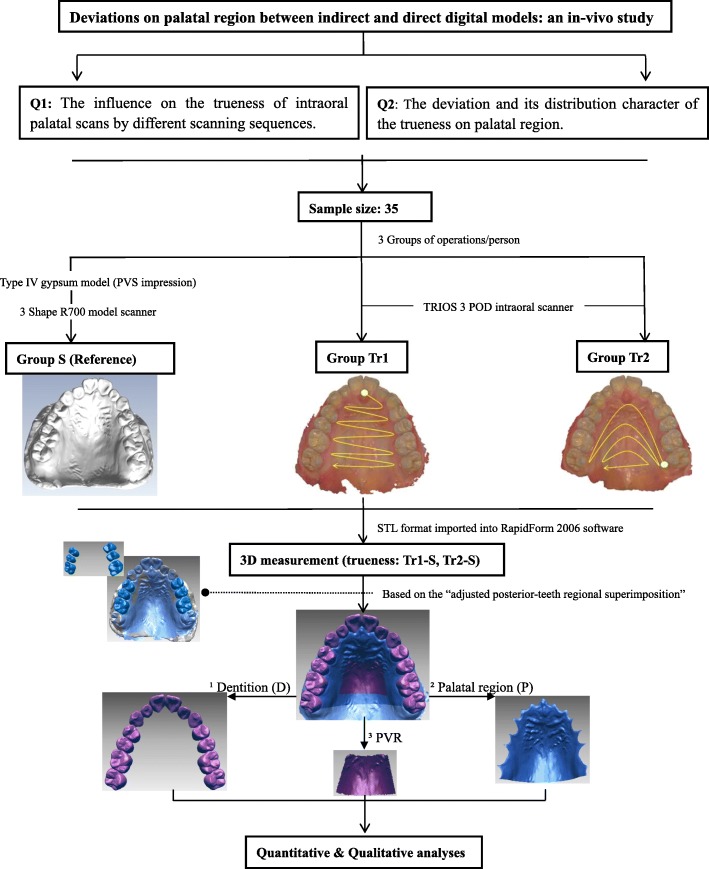
Flow diagram of the study

To acquire digital models of the upper jaws, each participant underwent three different processes as follows. Figure 1 presents a flow diagram of the same.

1. Group S: Indirect digital models were utilised as reference. Impressions were recorded using PVS material (DMG Silagum, Germany) by experienced operators in a one-step process, and cast models were fabricated using type IV gypsum (Heraeus, Germany). These plaster models were digitalised using a model scanner (3 Shape R700, Denmark), the accuracy of which is 20 μm [27].
2. Group Tr1: Direct intraoral scans of the upper jaws were performed using an intraoral scanner (3 Shape TRIOS 3 POD, Denmark). The upper dentition was scanned according to the recommended protocol [13], following the sequence of ^1^occlusal–^2^palatal–^3^buccal surfaces in a slow zigzag manner. The upper left second molar was set as the starting point for the initial scan. Whilst scanning palatal soft tissues, scanning was initiated from the palatal side of upper central incisors in a zigzag manner, in accordance with guidelines from a previous study [10].
3. Group Tr2: Direct intraoral scans of upper jaws were performed using the same intraoral scanner. The upper dentition was scanned using the same method as in Group Tr1. Palatal scanning was performed from palatal side of the upper second molar to palatal side of the opposite arch, finishing the entire palatal scanning at the distal end of the second molars by continuously narrowing down the scope in an inverted U manner. The palatal scanning sequence was similar to that described by Pavoni C et al. [28].

All intraoral scans were performed by one trained researcher. Re-scans were performed if any defects were identified with image stitching.

### 3D superimposition measurement

The models were imported in STL format into a 3D scan data metrology and modelling software (RapidForm 2006, INUS Technology, Inc., Korea) and were split into three parts for following measurements (Fig. 1).

1. Upper dentition (D): The upper dentition was selected by removing soft tissue along the gingival margin, except for existing third molars.
2. Palatal region (P): For selection of the palatal region, in view of characters of hard palatal anatomy, the distal boundary was set at the level of the mid-gingival margin of second molars. Models were trimmed by the orthogonal plane with horizontal and mid-sagittal planes at the distal boundary. The horizontal plane was created by the midpoints of gingival-margin of bilateral second molars and the point of gingival papilla of upper incisors. While, the mid-sagittal plane was perpendicular to the horizontal plane through the midline of palate. Subsequently, the palatal segment was selected along the gingival margin.
3. Palatal vault region (PVR): The palatal vault region was selected in accordance with borders suggested by Chen G et al. (2011) [25].

Subsequently, trueness of D, P, and PVR was separately measured. According to definition, trueness of intraoral scanning was determined by means of divergence between indirect and direct digital models in this study. Thus, trueness in the palatal region (P) in Group Tr1 was termed ‘Tr1-S (P)’, and so were the rest of the descriptions.

Various features of 3D measurement by different superimposition methods need to be discussed further.

#### 3D measurement by superimposition methods on different levels

Because scanning sequence in Group Tr1 was consistent with that reported in a previous study [10], trueness in Group Tr1 was analysed. Four types of registration levels were considered.

1. Adjusted dental-level: For 3D measurement of accuracy of intraoral digital impression in the palatal region, certain surfaces of non-free-end posterior teeth were selected for regional superimposition. This method was termed as ‘upper posterior-teeth regional superimposition (UPRS)’ (Fig. 1). Relatively accurate regions of dentition were selected for regional superimposition, namely buccal, occlusal and palatal surfaces of upper posterior teeth except the distal free-end teeth. Second molars were permitted if premolars were missing.

Reproducibility of this method was analysed by repeatedly selecting surfaces for secondary 3D measurement of trueness in the palatal region.

(2) Dental-level: The entire upper dentition was considered as a relatively accurate region for model superimposition.
(3) Palatal-level: Irrespective of the entire upper jaw, palatal regions were selected from the two models and directly superimposed.
(4) Upper jaw-level: Irrespective of differences in features, the entire upper jaws were globally superimposed.

**Fig. 2:**
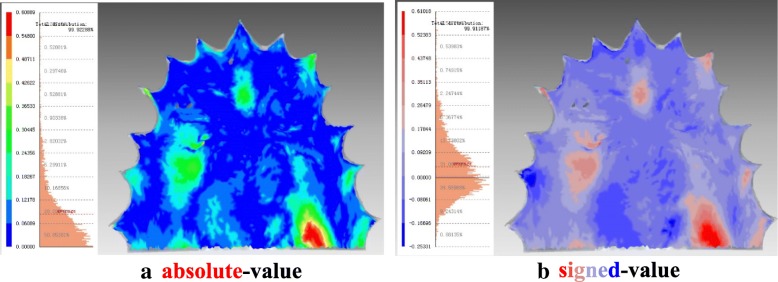
Representations of colour-coded maps. In an absolute colour map (**a**), blue indicates nearly no deviation and red indicates larger deviation. In a signed colour map (**b**), (+) expresses the local relation between shells of Groups Tr and S such that this part of Group S is located beneath and (−) reflects the reversed local relation

All 3D measurements were displayed in absolute-value and signed-value colour-coded maps (Fig. 2). Data from a calibrated scale was used for quantitative analyses, wherein mean absolute deviations were measured for trueness and mean signed deviations were analysed for distortion in shape.

#### Quantitative and qualitative analyses of trueness in the palatal region

Based on UPRS, trueness of intraoral digital models in the palatal region in Groups Tr1 and Tr2 was measured and statistically compared. In addition, trueness in PVR was measured and analysed independently. Moreover, colour maps were used for qualitative analyses, in an attempt to derive conclusions on deviation distribution/patterns of trueness of intraoral palatal scans.

### Statistical analysis

**Fig. 3:**
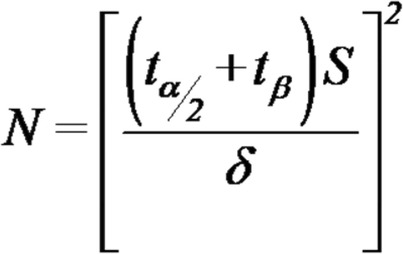
Formula of two-tailed paired t-test sample size

For quantitative analyses, data were statistically analysed using IBM SPSS Statistics (version 19, IBM Corp., USA). According to the formula (Fig. 3) [29], the sample size met the demand of paired t-test under the circumstances of α = 0.05 and power = 0.80, which required at least 32 participants. It was also sufficient for one-way analysis of variance (ANOVA), wherein the sample size per group was 14 subjects, as estimated using a statistical analysis software (PASS 11, NCSS, U.S.).

One-way repeated-measures ANOVA and Bonferroni post hoc test were used to analyse differences among the four superimposition methods in terms of trueness measurement for the palatal region. On the other hand, paired t test was used to analyse the influence of scanning sequences on palatal trueness and compare the trueness between palatal and dental regions. *p* values < 0.05 were considered statistically significant. Moreover, the intraclass correlation coefficient (ICC) was calculated to verify reproducibility of the superimposition in each region. The reproducibility of 3D measurements in absolute and signed-colour maps was represented by that of Palatal-level superimposition method.

## Results

### Participant characteristics

The study population included 9 men and 26 women, with an age range of 24–27 years. Seventeen participants had undergone orthodontic treatment, of whom, 7 had their premolars extracted. The mean arch width between bilateral upper first molars was 36.68 mm.

### Comparison of 3D measurement among different superimposition methods

3D measurements had satisfied reproducibility. ICCs for absolute-deviation and signed-deviation measurements were separately 1.000 (95% confidence interval [CI] 0.999, 1.000) and 0.901 (95% CI 0.805, 0.950).

Measured using four methods, trueness of intraoral digital models in Group Tr1 is summarised in Table 1. Based on ICCs, the reproducibility could be ordered as following: Palatal-level (1.000, 95% CI 0.999, 1.000) > Dental level (0.998, 95% CI 0.995, 0.999) > Upper Jaw-level (0.982, 95% CI 0.965, 0.991) > Adjusted-dental level (0.938, 95% CI 0.876, 0.969).

**Table 1:** Trueness of intraoral digital impression in Group Tr1 measured using different superimposition methods

	Absolute-value (μm)			Signed-value (μm)		
Methods	Palatal region	Dentition	*p* value	Palatal region	Dentition	*p* value
Adjusted-dental level	118.59 ± 37.67	69.22 ± 21.62	.000*	14.56 ± 93.32	35.26 ± 18.27	0.209
Dental-level	115.27 ± 36.03	64.92 ± 19.50	.000*	1.40 ± 90.38	24.09 ± 14.61	0.164
Palatal-level	80.48 ± 26.23	–	–	12.11 ± 25.40	–	–
Upper Jaw-level	94.89 ± 28.18	70.53 ± 22.33	.000*	7.55 ± 55.24	24.13 ± 26.26	0.210

*p values < 0.05 indicate statistical significance

**Fig. 4:**
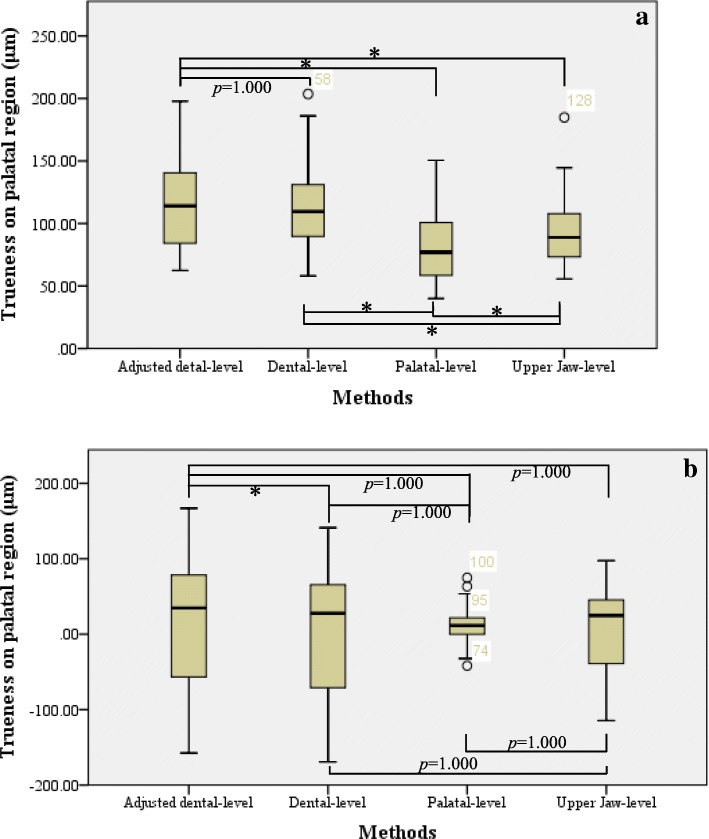
Comparison of trueness in palatal region in Group Tr1 between different superimposition methods. **a.** Absolute deviations of trueness in palatal region: in tests of within-subject effects, F (1.383, 47.009) =41.986, *p* < 0.0005, η^2^ = 0.553. **b.** Signed deviations of trueness in palatal region: in tests of within-subject effects, F (1.162, 39.506) =0.730, *p* = 0.418, η^2^ = 0.021. *Note:* Information shown in the boxplot are median, quartiles, minimum and maximum; the circle represents the outlier; asterisk (*) indicates statistical significance (*p* < 0.05)

By using one-way repeated-measures ANOVA, differences in trueness measurement for the palatal region measured using the aforementioned methods were analysed as follows:

1. For absolute deviations (Fig. 4-a), significant differences were identified among the four methods. By Bonferroni test, significant difference was noted among all methods (*p* < 0.0005), except for comparisons between adjusted dental-level and dental-level methods.
2. For signed deviations (Fig. 4-b), no significant difference was found among the four methods. Moreover, by Bonferroni test, no significant difference was noted among the methods, except for comparisons between adjusted dental-level and dental-level methods (*p* = 0.004 < 0.05).

**Fig. 5:**
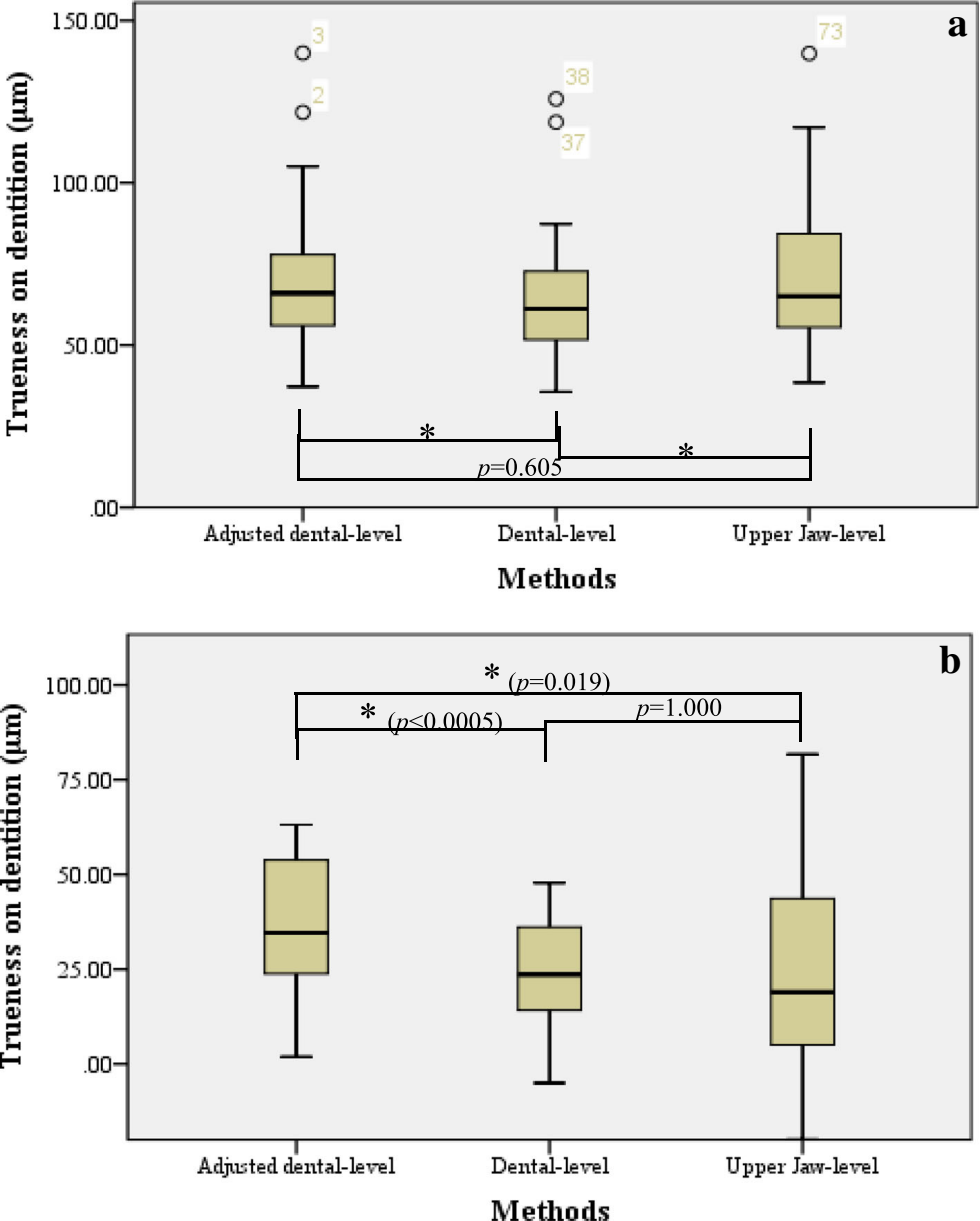
Comparison of trueness in dentition in Group Tr1 between different superimposition methods. **a.** Absolute deviations of trueness in dentition: in tests of within-subject effects, F (2, 68) =20.439, *p* < 0.0005, η^2^ = 0.375. **b.** Signed deviations of trueness in dentition: in tests of within-subject effects, F (1.270, 43.167) =8.971, *p* = 0.002, η^2^ = 0.209. *Note:* Information shown in the boxplot are median, quartiles, minimum and maximum; the circle represents the outlier; asterisk (*) indicates statistical significance (*p* < 0.05)

Furthermore, for 3D measurement of trueness in dentition, all methods except the palatal-level method were analysed using ANOVA:

(1) For absolute deviations (Fig. 5-a), significant differences were noted among all three methods. In addition, by Bonferroni test, significant differences were noted among each method (*p* < 0.0005), except for comparison between adjusted dental-level and upper jaw-level methods.

(2) For signed deviations (Fig. 5-b), significant differences were observed among all three methods. In addition, by Bonferroni test, significant differences were noted between each method, except for comparisons between dental-level and upper jaw-level methods.

For comparison of trueness between palatal region and dentition (Table 1), significant differences were noted in measurements of absolute deviations, while no significant difference was noted in measurements of signed deviations.

### Influence of different intraoral scanning sequences on palatal trueness

The influence of different scanning sequences on palatal trueness was investigated based on the UPRS method. Measurements of palatal trueness in Groups Tr1 and Tr2 are enlisted in Table 2.

**Table 2:** Comparison of palatal trueness between different intraoral scanning sequences measured using the UPRS method

*N* = 35	Absolute-value (μm)			Signed-value (μm)		
	Palatal region (P)	PVR	*p* value	Palatal region (P)	PVR	*p* value
Tr1-S (P)	118.59 ± 37.67	127.35 ± 54.11	0.129	14.56 ± 93.32	13.13 ± 126.00	0.898
Tr2-S (P)	108.25 ± 33.83	118.17 ± 49.52	0.092	18.01 ± 80.69	7.70 ± 112.76	0.373
*p* value	0.012*	0.149		0.582	0.615	

*p values < 0.05 indicate statistical significance

#### Comparison of palatal trueness between different scanning sequences

On comparing absolute deviations, deviations in Group Tr1 were larger than those in Group Tr2. The difference in mean values was 10.34 μm (95% CI 2.44, 18.25 μm) and was statistically significant (*p* = 0.012 < 0.05).

On comparing signed deviations, no significant difference was noted between the two groups (*p* = 0.582).

#### Comparison of PVR trueness between different scanning sequences

On comparing absolute deviations, no significant difference was noted between the two groups (*p* = 0.149). Moreover, compared with palatal trueness, the mean absolute deviation of trueness of PVR was larger without statistical significance. In Group Tr2, the difference in mean values between PVR and palatal trueness was 9.93 μm (95% CI − 1.72, 21.57 μm).

On comparing signed deviations, no significant difference was found between the two groups (*p* = 0.615). Moreover, compared with palatal trueness, the mean signed deviation of PVR trueness was smaller without statistical significance. In Group Tr2, the difference in mean values between PVR and palatal trueness was 10.32 μm (95% CI − 33.56, 12.92 μm).

## Patterns of deviation distribution of palatal trueness

### Characteristics of deviation distribution on the palate

**Fig. 6:**
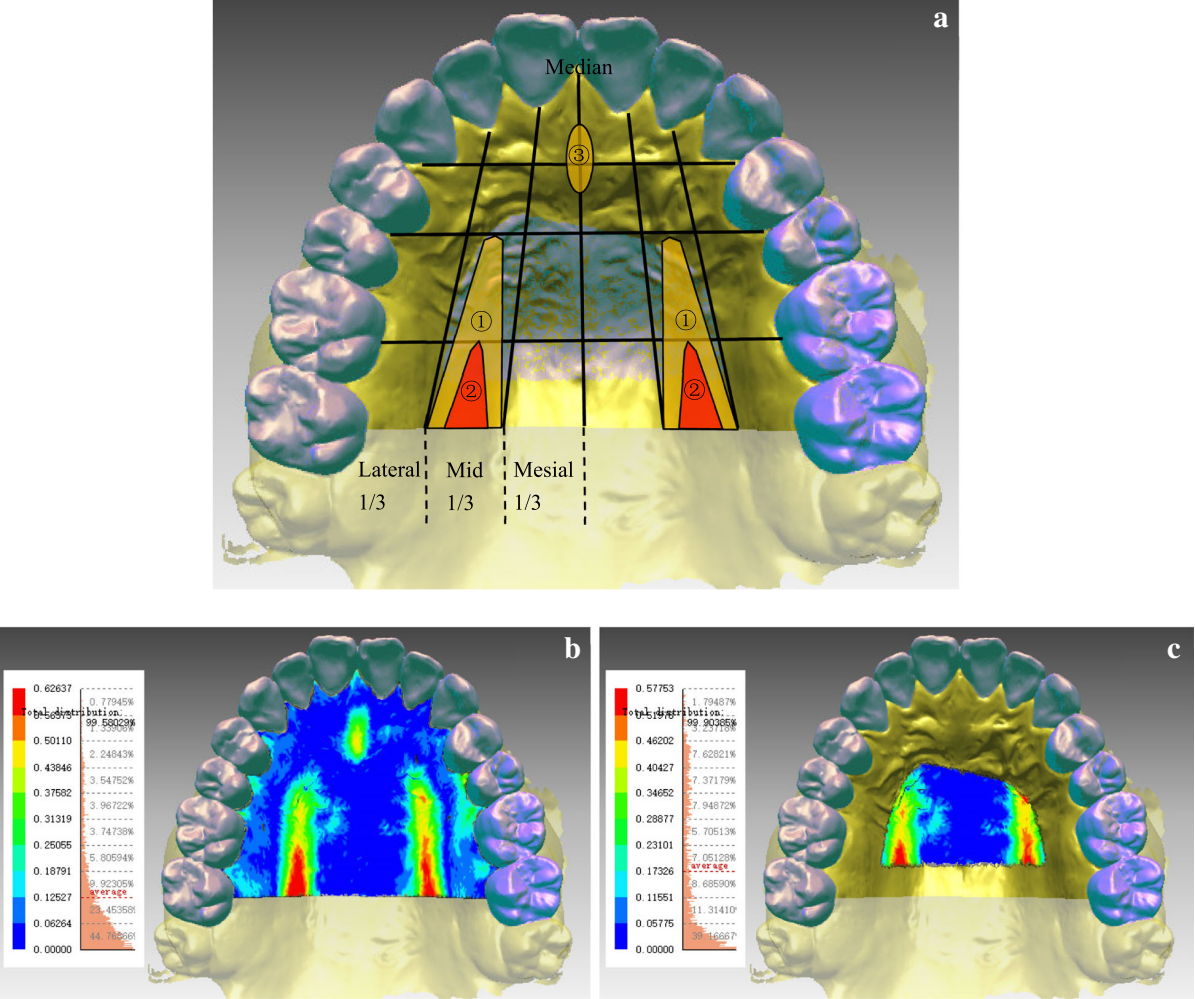
Pattern of palatal trueness of intraoral scans. **a.** Conceptual diagram of pattern of trueness in the palatal region. **b.** An illustration of the pattern of trueness in the palatal region. **c.** An illustration of the pattern of trueness in PVR

On observing colour-maps, a majority (22/35) of participants from both groups presented a similar pattern of palatal trueness (Fig. 6-a,b). Three local regions were more likely to present larger deviations, ordered by frequency and deviations as follows.

(i) Middle one-third of the palatal region at the level of second premolars and the distal part on unilateral or bilateral sides (Fig. 6-a①): the largest deviation usually appeared at the distal level of the mid-gingival margin of first molars (Fig. 6-a②). Largest deviations were typically ≥500 μm, whereas certain deviations were even close to 1000 μm.
(ii) Incisive papilla and the following part at the level between canines and first premolars (Fig. 6-a③): here, the deviations were seldom > 500 μm.
(iii) Gingival margin: deviations were irregularly distributed.

In addition, directions of larger deviations were almost positive. However, only a few cases (8/22) met all characteristics of the pattern.

Moreover, deviation distribution in the rest of the cases (13/35) was irregular and was noted at the level of first and second molars if there were obvious deviations. The deviations were typically ≤500 μm, except for one case in Group Tr1 with negative deviations ranging 485.91–813.95 μm over the left middle one-third of the palatal region at the level of first and second molars.

### Characteristics of deviation distribution on PVR

On lateral sides of PVR, it revolves part of middle one-third of the palatal region (Fig. 6-a,c). Therefore, larger deviations appeared at the sides of PVR, particularly those at the distal level of the mid-gingival margin of first molars, which could be > 500 μm and were usually positive. However, the same direction was not found for relatively small deviations.

## Discussion

### Methodology

Digital models can be acquired using three methods [29]: directly by intraoral scanning; impressions or plaster models digitalised using laboratory scanners; and digital models extracted directly from cone-beam computed tomography (CBCT) data or acquired using CBCT scans as well as laboratory scanners. Owing to radiation exposure and less accurate dental measurements [30, 31], the CBCT approach was not used in this study.

For 3D measurement of accuracy, the best method is model superimposition [4] with common use of a best-fit algorithm [15] as we did. Based on the normals technique [32, 33], 3D measurement was performed using the ‘shell-shell deviation’ function with high reproducibility.

A modified dental-level regional superimposition was proposed in this study. For participants who had undergone orthodontic treatment with premolar extraction, second molars were included in order to increase feature points for regional superimposition because the longer arch length, the larger the deviation of accuracy of dentition [7, 9]. Although the reproducibility was lower than that of the other three methods, according to the ICC value and its 95% confidence interval, the level of reliability of this method could be concluded as ‘good’ to ‘excellent’ [34].

Applications can be analysed using characters of superimposition and the measurements. (1) Both adjusted dental-level and dental-level methods are based on the prerequisite that the morphology of dentition in the intraoral digital impression is accurate [1–4]. When evaluating palatal trueness measured using these two methods, trueness in dentition should also be taken into consideration. Compared with the dental-level method, the adjusted dental-level method was more likely to represent distinct deviations and deformation on dentition (both with statistical significance). This maybe because there were more dental local deviations observed in intraoral digital impressions than in conventional impressions [2, 3]. Thus, it would highlight local deviations when relatively accurate regions were chosen for superimposition. (2) For the palatal-level method, the measurement of palatal trueness was the smallest. However, the outcome of this method cannot be extended to application in the entire upper jaw because of tendency of the best-fit algorithm to find a position with the least difference between two shells as well as less anatomical features on the palate compared with the dentition. (3) For the upper jaw-level method, it did not distinguish differences of surface characters and area between the palatal region and the dentition. Therefore, the most notable result of this method was that absolute deviations of trueness in the palatal region were statistically larger than those in the dentition. It was further verified that differences existed between trueness in the palatal region and the dentition, which was consistent with results reported by Gan N et al. [10].

Of note, when assessing palatal deformation (signed deviations) of intraoral digital impression, standard deviations (SDs) were distinctly higher than means, indicating that local deformation between the palatal region of intraoral digital models and plaster models was relatively unstable and irregular.

### Influence of different intraoral scanning sequences on palatal trueness

Because of more characters on posterior teeth and for image stitching based on additional feature points, in Group Tr2, palatal scanning was initiated on the palatal side of posterior teeth. Patterns of deviation distribution in both groups were similar in a majority of participants (22/35). The statistical difference in palatal trueness between Group Tr1 and Group Tr2 was primarily in values of larger deviations. Indeed, the process of image stitching is essential. The impact of scanning sequences on accuracy of dentition can be seen at regions with larger deviations; this results because of error accumulation during the process of image stitching [12, 13]. Palatal scanning in both groups was terminated at the molar level. Thus, the stitching error was converged at the distal end of the palate. Although there is no correlation between palatal trueness and arch width, deviations of palatal precision will increase with increasing arch width [10]. In Group Tr1, the entire palatal scan was finished by repeatedly scanning across the palate with increasing arch width. On the other hand, in Group Tr2, palatal scanning was finished by repeatedly scanning along the arch with decreasing scanning scope. Of note, the process of image stitching in Group Tr2 had three advantages: (i) there were more feature points at the beginning of palatal scanning; (ii) the possibility of deformation at palatal margins decreased by merging with the initial arch scan at the beginning of the palatal scan; and (iii) the scanning scope was decreasing in the process. It reduced the instability and error accumulation of scanning across the palate.

Regarding PVR, the region considered stable for model superimposition in orthodontics, there was no notable impact of different scanning sequences (Table 2). This indicates that it is reliable to use this region for superimposition of intraoral digital models regardless of which scanning sequences were performed. Above all, although the average difference of palatal trueness between the two scanning sequences was merely 10.34 μm, which is insufficient for it to be clinically significant. From the view of operation convenience and local deviations, we recommend the palatal scanning sequence in Group Tr2.

### Palatal trueness and its pattern of deviation distribution

Compared with findings from recent studies [10, 26], the measurement of palatal trueness in this study was smaller. This might be because selection of the studied palatal region was different from the aforementioned studies. In this study, the distal end was set at the level of the mid-gingival margin of second molars, with a mesial movement of half a tooth. The limitation of a plaster model was taken into consideration. (i) An in vitro study showed that there was slight deformation (50 μm) at the distal end of the arch (second molars) in plaster models [4], implying that some deformation of the palate might have occurred at the same level. (ii) Anatomically, there are more glands in the submucosa of the posterior region than that of the anterior region of the hard palate. In addition, greater palatine foramens are always located on the palatal side of third molars [35], where neurovascular bundles go through and then migrate mesially under the palatine mucoperiosteum. Therefore, the palatal shape at the level of second molars in conventional impression might be greatly affected by flexibility.

Regarding characteristics of deviation distribution of palatal trueness, findings were similar to that reported by Gan et al. [10], wherein positive deviations were noted at the palatal rugae and the two sides of the palatal vault. These regions and the direction of deviations implied that the uneven flexibility of palatal soft tissues during the impression procedure might lead to larger deviations. Moreover, whether the patterns were similar or not, regions with the largest deviations were consistent, namely the distal level of the mid-gingival margin of first molars, which was always the termination level of palatal scanning along with accumulation of scanning errors. However, nowadays, conventional impressions are still used as the reference, and hence, the pattern is interfered by the limitation of impact of flexibility.

### PVR trueness and its clinical significance

Compared with palatal trueness, the mean absolute deviation of PVR trueness was slightly higher, and the mean signed deviation of PVR trueness was closer to 0 μm with a larger discrete degree. This indicated that the area proportion of more obvious deformation in PVR was slightly higher than that in the entire palate. Regardless of scanning sequences, PVR trueness of 122.76 ± 48.51 μm was noted (95% CI 106.10, 139.42 μm). Although a deviation of > 500 μm is considered clinically significant [14], when assessing treatment changes by 3D PVR superimposition between plaster model and intraoral digital model, an error of 123 μm would make a difference with the development of Precision Medicine, such as a digital plan for teeth movement.

For 3D PVR superimposition [25], anterior and posterior boundaries of the selected region are the third palatal rugae and the distal end of first molars, respectively; lateral boundaries begin at the lateral one-third of the third palatal rugae and are parallel to the occlusal line of posterior teeth. By observation, larger deviations appeared at the lateral sides of PVR, particularly those at the distal level of the mid-gingival margin of first molars (Fig. 6-a,c). Therefore, although it would be reliable to use this region for superimposition of intraoral digital models, adjustments should be made when applying 3D PVR superimposition for plaster and intraoral digital models. In order to avoid the region of larger deformation and to maintain more distinctive structures, the distal boundary of the selected region for superimposition should be set at the level of the mid-gingival margin of first molars.

### Limitations

A major limitation of this study was the influence of flexibility of palatal mucosa on the reference model. To overcome this, selection of the palatal region was accordingly adjusted. However, from the viewpoint of characteristics of deviation distribution, the influence of flexibility during the impression procedure was still unavoidable. Another limitation was sex-imbalance in samples (9 males: 26 females). The area of the palate might be slightly larger in males. To counteract the possible effect of sex-imbalance on transversal and anterio-posterior dimensions of palate, it is better to balance sex distribution in samples. However, the statistical methods applied in this study were about self-control, less impacted by sex distribution. Furthermore, only one type of intraoral scanner was investigated in this study. In future, comparison among different intraoral scanners should be made to explore the divergency in reproducing palatal morphology.

## Conclusions

When evaluating accuracy of intraoral scanning in the palatal region, the superimposition method should be appropriately adjusted. Palatal trueness is affected by scanning sequences. We recommended that palatal scanning should be initiated at the palatal side of the posterior teeth where the initial scan begins, and the entire palatal scan should be finished along with the arch. Furthermore, when assessing treatment changes by using model superimposition of plaster and intraoral digital models, the average systematic 3D error was 123 μm. For 3D PVR superimposition, we recommend to adjust the distal boundary of the selected region mesially, at the level of the mid-gingival margin of first molars, whilst referring to intraoral digital models.

## Abbreviations

CBCT: Cone-beam computed tomography
ICC: Intraclass correlation coefficient
PVR: Palatal vault region
PVS: Polyvinyl-siloxane
STL: Standard tessellation language
UPRS: Upper posterior-teeth regional superimposition
VSE: Vinyl siloxanether
